# Elevated maternal lipoprotein (a) and neonatal renal vein thrombosis: a case report

**DOI:** 10.1186/1752-1947-2-106

**Published:** 2008-04-10

**Authors:** Vivek Subbiah, Prabhu Parimi

**Affiliations:** 1 Department of Internal Medicine/Pediatrics, Case Western Reserve University School of Medicine, MetroHealth Medical Center, Cleveland, OH 44109, USA; 2 Division of Neonatology, Department of Pediatrics, University of Kansas, KU Medical Center, Kansas City, KS 66160, USA

## Abstract

**Introduction:**

Renal vein thrombosis, although rare in adults, is well recognized in neonates and is one of the most common manifestations of neonatal thromboembolic events. The etiology of renal vein thrombosis remains unidentified in the majority of cases. We report a case of renal vein thrombosis in a neonate associated with elevated maternal lipoprotein (a).

**Case presentation:**

A full-term female infant, appropriate for gestational age, was born via spontaneous vaginal delivery to an 18-year-old primigravida. The infant's birth weight was 3680 g and the Apgar scores were eight and nine at 1 and 5 minutes respectively. Evaluation of the infant in the newborn nursery revealed a palpable mass in the right lumbar area. Tests revealed hematuria and a high serum creatinine level of 1.5 mg/dl. An abdominal ultrasound Doppler flow study demonstrated an enlarged right kidney, right renal vein thrombosis, and progression of the thrombosis to the inferior vena cava. There was no evidence of saggital sinus thrombosis. An extensive work-up of parents for hypercoagulable conditions was remarkable for a higher plasma lipoprotein (a) level of 73 mg/dl and an elevated fibrinogen level of 512 mg/dl in the mother. All paternal levels were normal. The plasma lipoprotein (a) level in the neonate was also normal. The neonate was treated with low molecular weight heparin (enoxaparin) at 1.5 mg/kg/day every 12 hours for 2 months, at which time a follow-up ultrasound Doppler flow study showed resolution of the thrombosis in both the renal vein and the inferior vena cava.

**Conclusion:**

There have been no studies to date that have explored the effect of abnormal maternal risk factors on fetal hemostasis. A case-control study is required to investigate whether elevated levels of maternal lipoprotein (a) may be a risk factor for neonatal thrombotic processes. Although infants with this presentation are typically treated with anticoagulation, there is a lack of evidence-based guidelines. Treatment modalities vary between study and treatment centers which warrants the establishment of a national registry.

## Introduction

Renal vein thrombosis (RVT), although rare in adults, is well recognized in neonates and is one of the most common manifestations of neonatal thromboembolic events [1]. The clinical signs of neonatal RVT (NRVT) include an enlarged kidney, hematuria, proteinuria, renal failure, hypertension and/or thrombocytopenia. Long-term consequences of NRVT include hypoplastic kidney, tubular defects, hypertension and renal insufficiency [2]. We report a case of NRVT associated with elevated maternal lipoprotein (a) [Lp (a)].

## Case presentation

**Figure 1:**
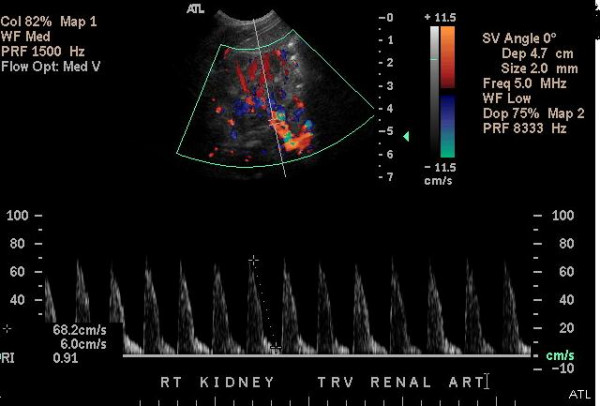
Renal Doppler flow study demonstrating an increased resistive index of the right renal artery with suboptimal wave forms of the right renal vein.

A full-term female infant, appropriate for gestational age, was born via spontaneous vaginal delivery, weighing 3680 g, to an 18-year-old primigravid Hispanic mother and a 21-year-old African American father. The neonate adapted well to extra-uterine life as evidenced by Apgar scores of eight and nine at 1 and 5 minutes, respectively. The pregnancy had been uneventful, and the maternal screens were all negative. There was no evidence of diabetes or pre-eclampsia during pregnancy. The neonate was transferred to the newborn nursery for routine newborn care. Physical examination in the newborn nursery revealed a palpable mass in the right lumbar area. Significant laboratory findings in the neonate included hematuria, and an elevated serum creatinine level of 1.5 mg/dl prompting transfer to the neonatal intensive care unit. A renal ultrasound evaluation showed an enlarged right kidney (5.56 cm) with loss of corticomedullary distinction. A renal Doppler flow study demonstrated an increased resistive index of the right renal artery with suboptimal wave forms of the right renal vein and a clot in the right renal vein (Figure 1). A Doppler flow study of the left renal artery and vein was normal. The neonate had normal blood pressures throughout the hospital stay.

The neonate and her parents were evaluated for prothrombotic risk factors. The mother's laboratory parameters were remarkable for an elevated level of Lp (a) of 73 mg/dl (normal 0 to 40 mg/dl), and fibrinogen of 512 mg/dl (156 to 400 mg/dl). The plasma Lp (a) concentration was measured by the enzyme-linked immunosorbent assay (ELISA) technique using mouse monoclonal anti-apo (a) capture antibody and sheep polyclonal anti-apoB detection antibody (COALIZA Lp (a), Chromogenix). The other laboratory tests on the mother were normal: plasma homocysteine 5.3 μmol/l; protein C activity greater than 125%; protein S activity greater than 125%; anticardiolipin antibody IgG 6.2 IgG phospholipid binding units; anticardiolipin IgM 6.4 IgM phospholipid binding units; beta 2 glycoprotein IgG less than 9 standard IgG antibeta 2 glycoprotein units; beta 2 glycoprotein IGM less than 9 standard IgM antibeta 2 glycoprotein units; lupus anticoagulant negative; Factor VIII A assay 108%; antinuclear antibody (ANA) screening negative; antithrombin III 94%; prothrombin time (PT), partial thromboplastin time (PTT), international normalized ratio (INR) normal. DNA analysis showed no point mutation (*G20210A*) in the 3' untranslated region of the prothrombin gene, no genetic polymorphism (*Arg 506/Glu 506*) for Factor V Leiden, and no gene mutation (*C677T*) for 5' 10 methylenetetrahydrofolate reductase (MTHFR). The paternal screens were all normal.

**Figure 2:**
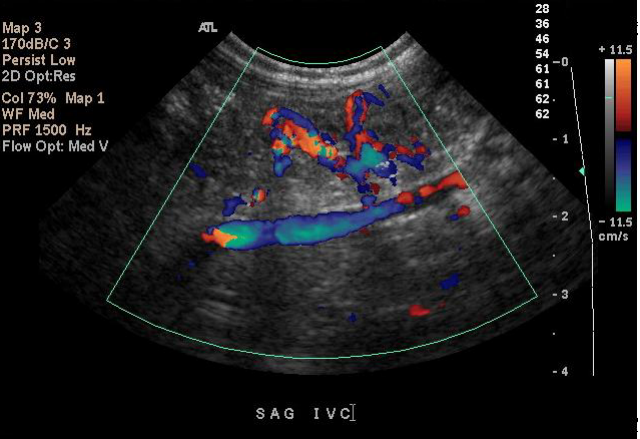
Follow-up renal Doppler ultrasound flow at 2 months of age showing the normal flow pattern of the inferior vena cava.

The plasma Lp (a) level in the neonate was 11 mg/dl (0 to 40 mg/dl). This was measured when the maternal Lp (a) results became available, that is, 4 days after the diagnosis of RVT. The plasma homocysteine level was 4.6 μmol/l (4 to 13.7 μmol/l). A computed tomography (CT) scan of the head of the neonate was negative for saggital sinus thrombosis. The neonate was started on low molecular weight heparin (enoxaparin) at 1.5 mg/kg/day every 12 hours, with antifactor Xa monitoring. The antifactor Xa activity measured was 0.74 IU/ml (less than 0.10 IU/ml). The serum creatinine level decreased to 0.5 mg/dl 9 days after initiation of treatment, and urinalysis showed no evidence of hematuria. Renal ultrasound Doppler flow studies were repeated at weekly intervals during the hospital stay. One week after initiation of treatment, an ultrasound demonstrated persistence of RVT and development of a new thrombosis in the inferior vena cava. The neonate was discharged home on enoxaparin and followed by a hematologist, a nephrologist and a primary care physician. A follow-up ultrasound Doppler flow study at 2 months of age showed a normal flow pattern in the aorta, inferior vena cava, right and left renal arteries and veins indicating resolution of the thrombosis (Fig. 2). Enoxaparin was discontinued at 2 months of age. The baby continued to do well with no evidence of residual renal dysfunction or hypertension at 18 months of age.

## Discussion

The etiology of NRVT remains unidentified in the majority of cases. The existence of underlying predisposing factors, such as asphyxia, sepsis, diabetic fetopathy or indwelling intravascular catheters, in combination with inherited prothrombotic risk factors, play a major role in the pathogenesis of NRVT [1-4], however their role is not well defined. The association between maternal thrombophilia and thrombotic complications in the neonate is unknown [5].

Lp (a) consists of phospholipids, cholesterol and apolipoprotein B-100 (low-density lipoprotein), with apolipoprotein (a) attached to the latter at a single point. Recent studies have demonstrated the significance of prothrombotic risk factors, especially the elevation of Lp (a) in the etiology of NRVT. It has been shown that Lp (a) competes with plasminogen for the plasminogen receptor on endothelial cells and initiates thrombosis. It has also been demonstrated that Lp (a) inactivates the 'tissue factor (TF) pathway inhibitor', which is a major endogenous regulator of TF-mediated coagulation [6]. Elevated plasma concentration of Lp (a) has been consistently shown to be a risk factor for the development of a variety of thrombotic and atherosclerotic disorders in humans. Lp (a) has been implicated in NRVT [1] as well as in cerebral venous thrombosis [5]. Lp (a) greater than 30 mg/dl has been shown to be a risk factor for the development of venous thromboembolism in children [7].

There is a paucity of data exploring prothrombotic risk factors in the development of NRVT in neonates. None of the studies reported to date have explored the effect of abnormal maternal risk factors on fetal hemostasis. The mechanism by which an elevated maternal Lp (a) with a normal level in the neonate contributes to the formation of a thrombus was unclear in this case. There is no evidence that Lp (a) crosses the placenta given the large size of this molecule. A higher level of maternal Lp (a) could be an independent risk factor in neonatal thromboembolic events. A case-control study is required to investigate whether elevated levels of maternal Lp (a) are a risk factor for neonatal thrombotic processes. In addition to measuring plasma levels of Lp (a) by standardized methods, genetic polymorphisms of Lp (a) should also be explored to identify secretor haplotypes.

## Conclusion

The infant reported here is now 18-months old, has normal renal function and has no evidence of hypertension. Although infants with this presentation are typically treated with anticoagulation, there is a lack of evidence-based guidelines. The treatment modalities vary between study and treatment centers which warrants the establishment of a national registry. Screening for prothrombotic risk factors in NRVT remains controversial [2,3,8,9]. Messinger et al. [3] have reported that all neonates with unilateral NRVT treated with heparin and with or without fibrinolytics had either small or atrophic kidneys between 2 and 6.5 years of age, but had normal renal function. This study underscores the importance of long-term follow-up of neonates with NRVT.

## Abbreviations

ANA: Antinuclear antibody; CT: Computed tomography; ELISA: Enzyme-linked immunosorbent assay; Lp (a): Lipoprotein (a); INR: International normalized ratio; MTHFR: 5' 10 methylene tetrahydrofolate reductase; NRVT: Neonatal renal vein thrombosis; PT: Prothrombin time, PTT: Partial thromboplastin time; RVT: Renal vein thrombosis; TF: Tissue factor.

## Competing interests

The authors declare that they have no competing interests.

## Authors' contributions

VS was involved in the patient's evaluation and clinical care, and in the conception of the report, literature review, and manuscript preparation, editing and submission. PP was involved in the patient's evaluation and clinical care, and in the conception of the report, manuscript preparation and editing, and administrative support. All authors have read and approved the final manuscript.

## Consent

Written informed consent was obtained from the patient's next-of-kin for publication of this case report and accompanying images. A copy of the written consent is available for review by the Editor-in-Chief of this journal.
